# Can machine learning assist in systemic sclerosis diagnosis and management? A scoping review

**DOI:** 10.1177/23971983241253718

**Published:** 2024-05-24

**Authors:** Eric P McMullen, Rajan S Grewal, Kyle Storm, Lawrence Mbuagbaw, Maxine R Maretzki, Maggie J Larché

**Affiliations:** 1 Michael G. DeGroote School of Medicine, McMaster University, Hamilton, ON, Canada; 2 School of Health, University of Waterloo, Waterloo, ON, Canada; 3 Department of Health Research Methods, Evidence and Impact, McMaster University, Hamilton, ON, Canada; 4 Cochrane Cameroon, Centre for Development of Best Practices in Health (CDBPH), Yaoundé Central Hospital, Yaoundé, Cameroon; 5 Biostatistics Unit, Father Sean O’Sullivan Research Centre, St Joseph’s Healthcare Hamilton, Hamilton, ON, Canada; 6 Department of Global Health, Stellenbosch University, Stellenbosch, South Africa; 7 Divisions of Rheumatology and Clinical Immunology and Allergy, Departments of Medicine and Pediatrics, McMaster University, Hamilton, ON, Canada

**Keywords:** Systemic sclerosis, scleroderma, treatment machine learning, artificial intelligence

## Abstract

This scoping review aims to summarize the existing literature on how machine learning can be used to impact systemic sclerosis diagnosis, management, and treatment. Following Preferred Reporting Items for Systematic reviews and Meta-Analyses extension for Scoping Reviews (PRISMA-ScR) reporting guidelines, Embase, Web of Science, Medline (PubMed), IEEE Xplore, and ACM Digital Library were searched from inception to 3 March 2024, for primary literature reporting on machine learning models in any capacity regarding scleroderma. Following robust triaging, 11 retrospective studies were included in this scoping review. Three studies focused on the diagnosis of scleroderma to influence preferred management and nine studies on treatment and predicting treatment response to scleroderma. Nine studies used supervision in their machine learning model training; two used supervised and unsupervised training and one used solely unsupervised training. A total of 817 patients were included in the data sets. Seven of the included articles used patients from the United States, one from Belgium, two from Japan, and two from China. Although currently limited to retrospective studies, the results indicate that machine learning modeling may have a role in early diagnosis, management, therapeutic decision-making, and in the development of future therapies for systemic sclerosis. Prospective studies examining the use of machine learning in clinical practice are recommended to confirm the utility of machine learning in patients with systemic sclerosis.

## Background

Systemic sclerosis (SSc) is an autoimmune connective tissue condition resulting in the fibrosis of skin and internal organs including the gastrointestinal tract, heart, lungs, and kidneys.^1^

The etiology of SSc is not known, with a complex triad of vasculopathy, immune dysregulation, and fibrosis, causing damage in skin and multiple organs. No curative treatments exist.^2^ Therapies aim to relieve symptoms and slow disease progression in the affected organs. However, even recent advances such as immunomodulatory therapies are not curative, and treatment effects are limited once fibrosis is established. Hematopoietic stem cell transplantation (HSCT) has been used in severe cases. This treatment seeks to halt disease progression by transfusing undifferentiated stem cells allowing regeneration of the affected fibrotic tissue and improvement in functional outcomes.^3^ While this treatment has proven to be effective in treating severe disease, it is not recommended for mild cases due to the invasive nature of the therapy and the risk of serious adverse events.^3^

Artificial intelligence (AI) is defined as a machine-based system that aims to make predictions, recommendations, and decisions emblematic of the ability to learn (i.e. service knowledge from data).^4^ In the field of medicine, it is expected that AI will work alongside electronic medical records (EMRs) to assist in predictions about health outcomes as the technology develops. AI technology “learns” by training with thorough data sets that contain a vast amount of patient information like vitals, genetics, and environment.^4^ Machine learning (ML) is a subtype of AI that “learns” using prediction models and algorithms to analyze complex data patterns. In the healthcare field, this technology is most often seen in precision medicine, in which treatment decisions are based on predictions made by an ML model that has examined a variety of patient attributes and the context behind the treatment.^4^

With the complexity of SSc, ML can be a useful approach in understanding the manifestations of the disease and its management. While tools like composite diagnostic criteria such as ACR/EULAR 2012 classification criteria have been used to confirm diagnosis and the modified Rodnan Skin Score (mRSS) and Composite Response Index in Systemic Sclerosis (CRISS) scores used to determine activity and extent of disease have been used historically to determine extent and activity of SSc, advances in ML tools have the potential to improve accuracy in diagnostics, prognostication, and analysis of treatment outcomes.^5–8^ Furthermore, ML could identify potential new therapies in this disease. The extent to which ML has been used in SSc diagnosis, management and outcomes research is not known. It is also unclear which areas of SSc research would benefit most from ML techniques. An improved understanding of which ML approaches have been used in SSc is needed to improve the understanding and management of this disease. The objective of the current review is to summarize the existing literature on how ML can be used to impact SSc management with applications such as in assessing treatment response, along with SSc diagnosis and severity in influencing preferred management.

## Methods

The Preferred Reporting Items for Systematic reviews and Meta-Analyses (PRISMA) checklist for scoping reviews was used to inform the reporting of this scoping review (Supplementary File 1).^9^

### Inclusion and exclusion criteria

English primary peer-reviewed literature of any type that applied ML models to SSc treatment was included. Articles were excluded if they did not involve SSc treatment, or if ML was not included in the study methods. Conference abstracts were excluded due to their brevity.

### Information sources

Embase, Web of Science, Medline (PubMed), Scopus, IEEE Xplore, and ACM Digital Library were searched from inception to 3 March 2024. Forward and backward citation analysis of relevant records was performed using Web of Science to identify potentially eligible studies as well.

### Search strategy

The search terms “scleroderma,” “systemic sclerosis,” and a variety of ML-related search terms were used. We did not use the search terms, “treatment,” or “therapeutic” as it would unnecessarily narrow our search (Table S1, Supplementary File 1). Duplicates were removed, and all types of primary literature aligning with the above inclusion and exclusion criteria were eligible in initial title and abstract screening. Relevant articles moved on to full-text review, and citation chaining of included articles was performed. Articles were imported into the systematic review software, Covidence.^10^

Two reviewers (R.S.G. and K.S.) completed the initial title and abstract screening and full-text screening independently and in duplicate. Any disagreements were settled through discussion with a third author (E.P.M.). Data were also extracted independently and in duplicate. Conflicts were settled through review by E.P.M.

### Data charting and result reporting

Data were extracted from the included articles into a data extraction table on an Excel spreadsheet, piloted with the first 10 studies to ensure that the categories matched the goals of extraction. The extraction table was created by E.P.M. and reviewed by R.S.G. The following study-related variables were collected: author(s), year of publication, country, study aim, type of ML model used, specific ML model used, reference test used, population (n), treatment used, conclusions, and reported study outcomes.

## Results

**Figure 1.**
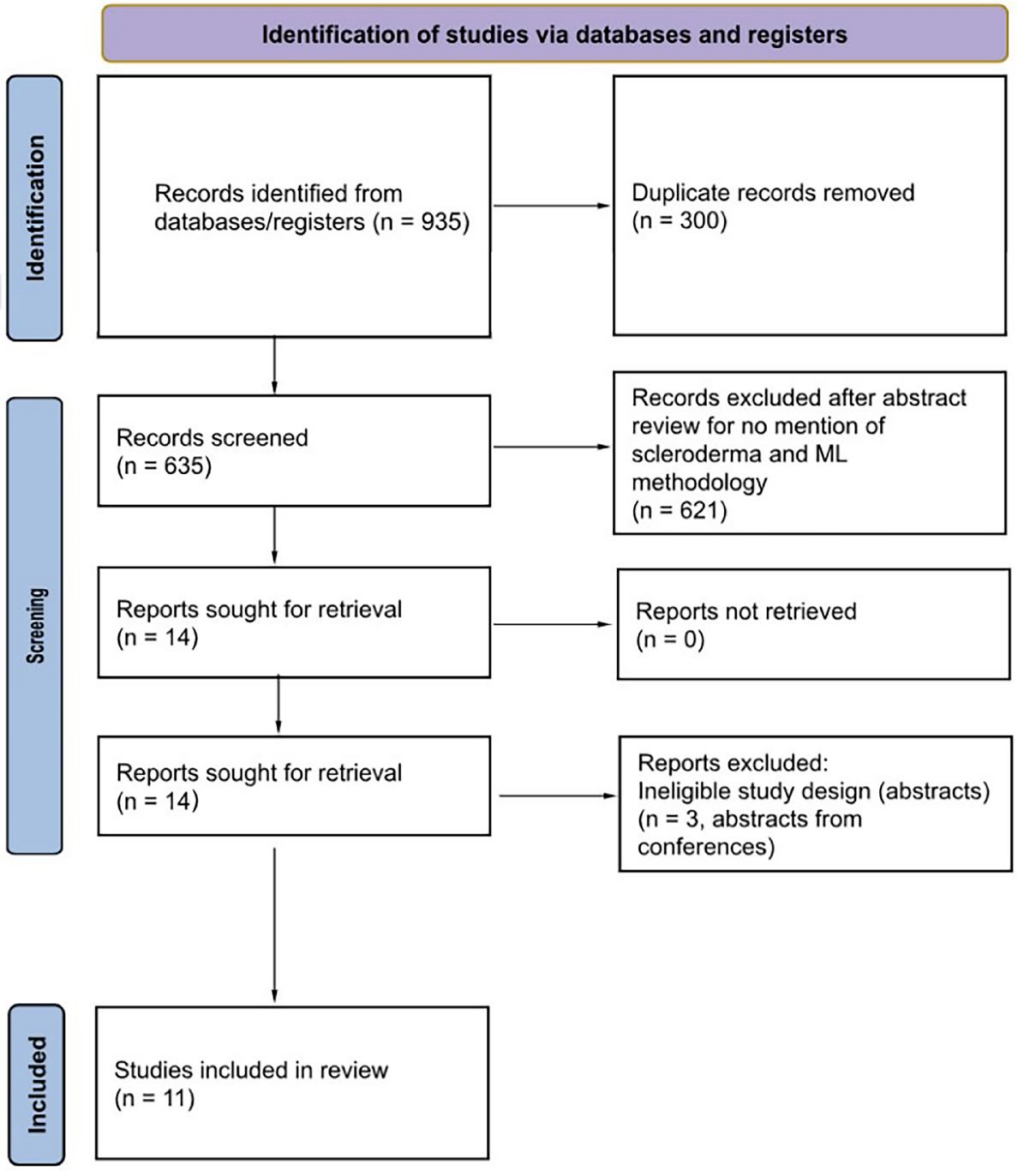
Flow diagram of literature screening according to the Preferred Reporting Items for Systematic reviews and Meta-Analyses extension for Scoping Reviews (PRISMA-ScR) guidelines.

The initial electronic search returned 935 articles. After duplicates were removed, 635 articles advanced to title and abstract screening. 621 were excluded, leaving 14 to move on to full-text review. Reasons for exclusion at the full-text stage are reported (Figure 1). Overall, a total of 11 articles fulfilled the search criteria and were included in our scoping review (Figure 1). The most frequently used ML models were GLMnet, random forest (RF), and support vector machine (SVM) (Table 2).

### ML applications on SSc diagnosis and severity to influence preferred management

Four studies focused on attempting to classify and diagnose SSc (Table 1).^5,11–13^ Between the studies, the accuracy in comparison to conventional assessment ranged from 85.4% to 87.7% in validation image sets.^11,12^ In one study, ML was used to assign histological images of SSc to subsets of disease and correlate them to candidate genetic biomarkers to stratify the severity of disease.^5^ They showed that CD34 and aSMA may be relevant biomarkers in predicting both disease severity and improvement over time.^5^ One of these studies used a similar method with RF and GLMnet to achieve the same goal of identifying SSc interstitial lung disease progression from a set of high-resolution computed tomography (HR-CT) images.^13^

**Table 1.** Summary of articles reporting on ML applications of scleroderma diagnosis and severity to influence preferred management.

Authors, country	Study design	Aim	Type of AI methodology used	Specific AI methodology used	Reference test	Population (n)	Treatment	Conclusions
Akay et al.,^11^ The United States	Parallel RCT	To develop a CNN for the purpose of early diagnosis of SSc	Supervised	MobileNetV2, a CNN	Scores classified by AI compared against data gathered from clinician diagnoses	40	N/A	The proposed network architecture could be implemented in clinical settings, providing a simple, inexpensive, and accurate screening tool for SSc
Franks et al.,^12^ The United States	Proof of concept	To introduce a novel machine learning classifier as an accurate predictor of intrinsic subsets of SSc	Supervised and unsupervised	RF and GLMnet used to train supervised classifiers. SVM trained with a linear kernel	N/A	213	N/A	Developed highly accurate classifier for SSc molecular subsets for individual patient samples. Method can be applied in SSc clinical trials to identify intrinsic subsets on individual samples
Kim et al.,^13^ The United States	To examine changes in extent of patterns of ILD during immunosuppressive therapy in SSc-ILD	Supervised	SVM to analyze the ILD patterns	N/R	N/A	97	CYC for 1 year followed by placebo for additional year or MMF for 2 years	Immunosuppressive treatment for SSc-ILD is effective in restoring a normal lung pattern in patients with both ground-glass and lung fibrosis ILD patterns
Showalter et al.,^5^ The United States	Parallel RCT	To determine histologic and gene expression features of clinical improvement in early diffuse cutaneous SSc	Supervised	GLMnet used to assign samples to gene expression subsets. SVM to determine histologic features predictive of gene expression subset	N/A	26	N/A	CD34 and aSMA stains may be useful biomarkers of clinical severity and improvement in dcSSc

RCT: randomized controlled trial; CNN: convolutional neural network; SSc: systemic sclerosis; N/A: not applicable; mRSS: modified Rodnan skin score; SVM: support vector machine; RF: random forest; SCOT: scleroderma cyclophosphamide or transplantation; HSCT: hematopoietic stem cell transplant; AI: artificial intelligence; CYC: cyclophosphamide; ILD: interstitial lung disease; MMF: mycophenolate mofetil; DML: debiased machine learning; PFT: pulmonary function tests; SSc-PAH: systemic sclerosis–assisted pulmonary arterial hypertension; XGBOOST: extreme gradient boosting algorithm.

### ML applications on SSc treatment

Seven studies reported ML methodology pertaining to the treatment of SSc (Table 2).^3,14–19^ Although the measured outcomes were different for each of the studies, there was significant overlap in the types of treatments that were examined. The main therapies among the included articles were immunosuppressants such as rituximab and mycophenolate, HSCT for more severe cases, and vasodilators (Table 2).

**Table 2.** Summary of findings reporting on ML applications on scleroderma treatment and treatment response.

Authors, country	Aim	Type of AI methodology used	Specific AI methodology used	Reference test	Population (n)	Treatment	Conclusions
Ebata et al.,^14^ Japan	To identify SSc patients suitable for treatment with rituximab and to determine appropriate treatment strategy for each patient	Supervised	Decision tree to predict the rituximab effect on improvement of mRSS in SSc	Four doctors who received standardized mRSS training; mRSS scoring was done by one doctor at each facility	54	Rituximab	Made decision tree predicting rituximab effect on improvement of mRSS in SSc. Findings suggest that of all combinations of patient biomarker with rituximab treatment response, SSc patients with high peripheral blood B-cell counts and high mRSS were the best candidates for rituximab therapy.
Franks et al.,^3^ The United States	To test hypothesis that peripheral blood cell sample from SCOT clinical trials predict long-term response to HSCT	Supervised	GLMnet to assign sample to intrinsic subset according to relative expression levels of large set of genes	Scores classified by AI compared against data gathered from clinicians in previous studies	63	CYC or HSCT	Study suggests that intrinsic subset patient stratification may be used to identify patients with SSc who receive significant benefits from HSCT.
Mehta et al.,^15^ The United States	To analyze efficacy of abatacept in patients with early dcSSc to test hypothesis that patients in inflammatory intrinsic subset would show most significant clinical improvement	Supervised	SVM classifier used to call molecular subsets	Scores classified by AI compared against data gathered from clinicians in previous studies	233	Abatacept	SSc inflammatory subset patients showed increased abatacept-targeted pathway expression, benefiting most with clinical improvement and skin fibrosis resolution.
Ninagawa et al.,^16^ Japan	To predict the response to pulmonary vasodilators in patients with SSc and PAH	Supervised	ResNet-50 was used as a CNN	N/R	84	Vasodilators	The predicted value of abnormal lung volume was superior to forced vital capacity for the response to vasodilators (AUC 0.74).
Wang et al.,^17^ China	To clarify the compound–gene relationship in subtypes of SSc to ultimately inform clinical treatment decisions	Unsupervised	SVM, RF, Boruta, and LASSO algorithms were used to select feature genes. Diagnostic models were created using SVM, RF, and LR	N/R	N/R	All compounds in the Comparative Toxicogenomics Database	The study identified eight feature genes of which eight compounds (methotrexate, resveratrol, paclitaxel, trichloroethylene, formaldehyde, silicon dioxide, benzene, and tetrachloroethylene) were identified to interact both positively and negatively with them.
Yan et al.,^18^ China	To identify hub genes, diagnostic markers and explore potential small-molecule drugs of SSc	Unsupervised	LASSO regression algorithm employed to screen prognostic SSc-related signature genes	N/R	N/R	Small molecule drugs	Ten small-molecule drugs with potential therapeutic effects were identified, mainly including phosphodiesterase (PDE) inhibitors (BRL-50481, dipyridamole), TGF-β receptor inhibitor (SB-525334), etc.
Zamanian et al.,^19^ The United States	To investigate the safety and efficacy of B-cell depletion for SSc-PAH, using change in 6-min-walk distance as the outcome measure	Supervised	RF, XGBOOST, and SVMs with polynomial and radial kernel transformations were used	N/R	57	Rituximab	B-cell depletion therapy is a potentially effective and safe adjuvant treatment for SSc-PAH. The XGBOOST algorithm outperformed three alternative machine learning algorithms.

RCT: randomized controlled trial; CNN: convolutional neural network; SSc: systemic sclerosis; N/A: not applicable; mRSS: modified Rodnan skin score; SVM: support vector machine; RF: random forest; SCOT: scleroderma cyclophosphamide or transplantation; HSCT: hematopoietic stem cell transplant; AI: artificial intelligence; CYC: cyclophosphamide; ILD: interstitial lung disease; MMF: mycophenolate mofetil; DML: debiased machine learning; PFT: pulmonary function tests; SSc-PAH: systemic sclerosis–assisted pulmonary arterial hypertension; XGBOOST: extreme gradient boosting algorithm.

A total of five articles directly studied the use of immunosuppressive and immunomodulatory medication in the treatment of SSc.^3^,^12–15^ Due to the high level of variability in the performance metrics of the treatment response studied, it is difficult to draw direct comparisons between the performance of the ML models used in each study. Regardless, the results of immunosuppressive therapies in improving SSc demonstrated effectiveness in improving disease outcomes. One study indicated that patients with high B-cell counts respond most positively to rituximab therapy, with those with low baseline B-cell counts not responding.^14^ This efficacy of B-cell depletion therapy was replicated in another study of SSc-induced pulmonary arterial hypertension.^19^ A significant improvement in 6-min walk distance after 24 weeks of 25.5 ± 8.8 m for the rituximab group and only 0.4 ± 7.4 m for the placebo group was reported.^19^

One study examined the use of vasodilators in the treatment of SSc pulmonary hypertension and through the use of a convolutional neural network (CNN) found that calculated abnormal lung volume was superior to forced vital capacity for predicting response to vasodilators (area-under-the-curve (AUC) = 0.74).^16^

Two recent studies from 2023, examined SSc treatment, through the lens of genetic analysis.^17,18^ Examining the compound–gene relationship in subtypes of SSc, eight feature genes along with associated potential treatments (methotrexate, resveratrol, paclitaxel, trichloroethylene, formaldehyde, silicon dioxide, benzene, and tetrachloroethylene) were identified.^17^ Another study identified hub genes, and other diagnostic markers. Seven hub genes that may play a role in SSc pathways and development were identified including THY1 and SULF1 as prognostic markers.^18^ Ten small-molecule drugs with potential therapeutic effects were also identified, mainly including phosphodiesterase inhibitors (BRL-50481, dipyridamole) and transforming growth factor beta (TGF-β) receptor inhibitors (SB-525334).^18^

### Accuracy of included ML models

Specific data points surrounding the effectiveness of each ML methodology in the included studies were sparse. One study provided an AUC value for their ML methodologies, finding, and AUCs = 0.74.^16^ Extreme gradient boosting algorithm (XGBOOST) was the most reliable methodology compared to the three others (RFs, SVM polynomial, and SVM radial) trained, validated, and tested.^19^

## Discussion

The results of this review demonstrate the various positive applications of ML in clinical practice relating to SSc. ML tools have the potential to be implemented alongside existing tools such as the composite measures to diagnose patients with SSc. Improving the speed and accuracy of diagnoses may improve patient prognosis. Furthermore, since SSc has variable systemic manifestations, early detection will prompt improved monitoring for critical organ damage and inform earlier treatment decisions. All of the studies in this review were able to utilize ML in promising ways. While most of the models assisted in the measurement of treatment outcomes, others achieved a high level of accuracy in identifying SSc subtypes. ML modeling may have a role in the identification and development of future therapies for SSc and may be an effective tool for clinicians to predict treatment outcomes.

There was significant variation in the chosen performance metrics among the included articles, and for this reason, they could not be analyzed. Due to the limited body of research, more studies are needed to determine which models are best for unique purposes. Only one of the included studies directly compared multiple ML models in carrying out their research. On comparing four different ML models against each other, XGBOOST outperformed the other models by a significant margin.^19^

While the results of this review are promising, the review is not without limitations. Including only peer-reviewed, articles written in English, likely led to an overrepresentation of research from English-speaking countries. Moreover, it is likely that many of the studied populations were lacking in ethnic diversity. While most of the included articles opted not to report statistics on the ethnicities of the participants, white individuals are highly overrepresented in those that did. For example, in one study, white individuals comprised 76.7% of the study participants.^3^ There is some evidence to suggest that individuals of different ethnic backgrounds experience autoimmune diseases, particularly SSc, in varying degrees of severity.^20^ Thus, a study design that examines a more diverse population would likely provide more comprehensive results.

There was a high degree of variability between the objectives and outcomes of each included study. This could be attributed to the novelty of the field, with the majority of research on this topic published in the last few years. As more studies emerge, the heterogeneity will likely decrease.

Finally, none of the reviewed ML models were implemented prospectively in clinical practice. This is a major limitation in assessing the utility of ML in diagnosis, prognosis, and optimal management of SSc. However, the majority of these studies reported their goal of implementing ML models to improve clinical practice in the future.

## Conclusion

Research examining the applications of ML in SSc is rapidly developing and has received significant attention in recent years. This scoping review shows that in supervised settings, several ML models have demonstrated a high level of accuracy in identifying SSc at early stages, and in classifying severity and measuring disease progression over time. Response to treatment and identification of molecules that may improve disease outcome have also been shown to be reasonable outcomes from these studies; however, these studies are retrospective in design. As the literature grows, ML can gradually be implemented into clinical practice for the analysis of empirical data to augment clinicians’ ability to diagnose, assess, manage, and predict treatment response. Future research should focus on investigating the utility of ML modeling compared to traditional methods in managing SSc cases. Despite the promising results, the lack of homogeneity among existing studies creates difficulties in drawing informed conclusions about the current applications. Further research with larger data sets comparing a variety of ML models would provide a greater body of evidence to draw from and is recommended for a clearer assessment.

## Supplemental Material

sj-pdf-1-jso-10.1177_23971983241253718 – Supplemental material for Can machine learning assist in systemic sclerosis diagnosis and management? A scoping review Supplemental material, sj-pdf-1-jso-10.1177_23971983241253718 for Can machine learning assist in systemic sclerosis diagnosis and management? A scoping review by Eric P McMullen, Rajan S Grewal, Kyle Storm, Lawrence Mbuagbaw, Maxine R Maretzki and Maggie J Larché in Journal of Scleroderma and Related Disorders
