# Supplementary material for: Can machine learning assist in systemic sclerosis diagnosis and management? A scoping review
Source: J Scleroderma Relat Disord. 2024 May 24;9(3):171–7. doi: 10.1177/23971983241253718 (PMC11528611; doi:10.1177/23971983241253718)
Supplement: sj-pdf-1-jso-10.1177_23971983241253718 – Supplemental material for Can machine learning assist in systemic sclerosis diagnosis and management? A scoping review [file sj-pdf-1-jso-10.1177_23971983241253718.pdf]

Supplemental File:

Scleroderma therapy through machine learning-based strategies: A scoping review

Supplementary Table S1. Search strategy.

Databases:
Embase <1974 to 2024 March 03>
Ovid MEDLINE(R) ALL <1946 to March 03, 2024>

#	Query	Results from 3 Mar 2024
1	exp artificial intelligence/	288,061
2	exp machine learning/	522,441
3	deep learning/	71,431
4	((artificial* or machine* or deep*) adj3 (intelligence or learning)).tw,kw.	366,755
5	AI.ti,ab.	120,976
6	computer* assist* diagnosis.tw,kw.	2,045
7	computer vision.mp.	19,666
8	supervised learn*.mp.	13,522
9	neural network*.mp.	271,647
10	unsupervised learn*.mp.	6,284
11	natural language process*.mp.	26,758
12	segmentat*.mp.	149,516
13	reinforcement learn*.mp.	14,775
14	exp scleroderma/	55,614
15	scleroderma.mp.	63,580
16	systemic sclerosis.mp.	59,639
17	exp systemic sclerosis/	61,653
18	14 or 15 or 16 or 17	94,900
19	1 or 2 or 3 or 4 or 5 or 6 or 7 or 8 or 9 or 10 or 11 or 12 or 13	1,083,084
20	18 and 19	640

Web of science (all databases): 256 results
("scleroderma" OR "systemic sclerosis") AND ("machine learning" OR "artificial intelligence" OR "deep learning" OR "computer vision" OR "neural network" OR "supervised learning" OR "unsupervised learning" OR "natural language processing" OR "segmentation" OR "reinforcement learning")

IEEE Xplore: 14 results
("scleroderma" OR "systemic sclerosis") AND ("machine learning" OR "artificial intelligence" OR "deep learning" OR "computer vision" OR "neural network" OR "supervised learning" OR "unsupervised learning" OR "natural language processing" OR "segmentation" OR "reinforcement learning")

ACM Digital Library: 25 results
("scleroderma" OR "systemic sclerosis") AND ("machine learning" OR "artificial intelligence" OR "deep learning" OR "computer vision" OR "neural network" OR "supervised learning" OR "unsupervised learning" OR "natural language processing" OR "segmentation" OR "reinforcement learning")
